# Case report: Nocardia farcinica pneumonia in early-stage post liver transplantation

**DOI:** 10.3389/fmed.2022.996045

**Published:** 2022-09-07

**Authors:** Bing Pan, Fang-Fei Wang, Qiang He

**Affiliations:** Department of Hepatobiliary Surgery, Beijing Chaoyang Hospital, Capital Medical University, Beijing, China

**Keywords:** liver transplantation, pneumonia, Nocardia farcinica, microbiological tests, TMP-SMX, imaging

## Abstract

**Background:**

Liver transplantation is a well-established treatment for end-stage liver disease. The evolution of immunosuppressants has supported the recent advances in this field. However, this leads to immunosuppression and increases the risk for infections. Nocardia is an aerobic gram-positive bacillus, which can cause multi-systemic or multi-organ infections. Nocardia is an opportunistic pathogen that principally affects immunosuppressed patients.

**Case presentation:**

Herein, we present a case of Nocardia farcinica pneumonia in a patient at early-stage post-liver transplantation. Following appropriate microbiological tests and imaging, the diagnosis was finally confirmed. A full recovery was achieved after optimal antibiotic therapy of sulfamethoxazole, minocycline, and amikacin.

**Conclusions:**

Nocardia farcinica pneumonia is a rare and life-threatening disease, especially in patients after liver transplantation. Imaging and microbiological tests are helpful for the early diagnosis of the disease. Trimethoprim-sulfamethoxazole (TMP-SMX) as part of first-line therapy for nocardiosis is recommended.

## Introduction

Liver transplantation is a well-established treatment for end-stage liver disease (1). The evolution of immunosuppressants has supported the recent advances in this field. However, this leads to immunosuppression and increases the risk for infections (2). Nocardia is an aerobic gram-positive bacillus, which can cause multi-systemic or multi-organ infections. Nocardia is an opportunistic pathogen that principally affects immunosuppressed patients (3). Nocardia farcinica, accounting for 24.5% of all Nocardia infections, is more likely to cause disseminated infection and higher mortality (4). However, Nocardia has received relatively little attention as a human pathogen. It is difficult to detect and diagnose and leads to delays in medical treatment and poor prognosis. Herein, we present a case of Nocardia farcinica pneumonia in a patient at early-stage post liver transplantation in our department. Following appropriate microbiological tests and imaging, the diagnosis was finally confirmed. A full recovery was achieved after optimal antibiotic therapy of sulfonamide.

## Case presentation

**Figure 1:**
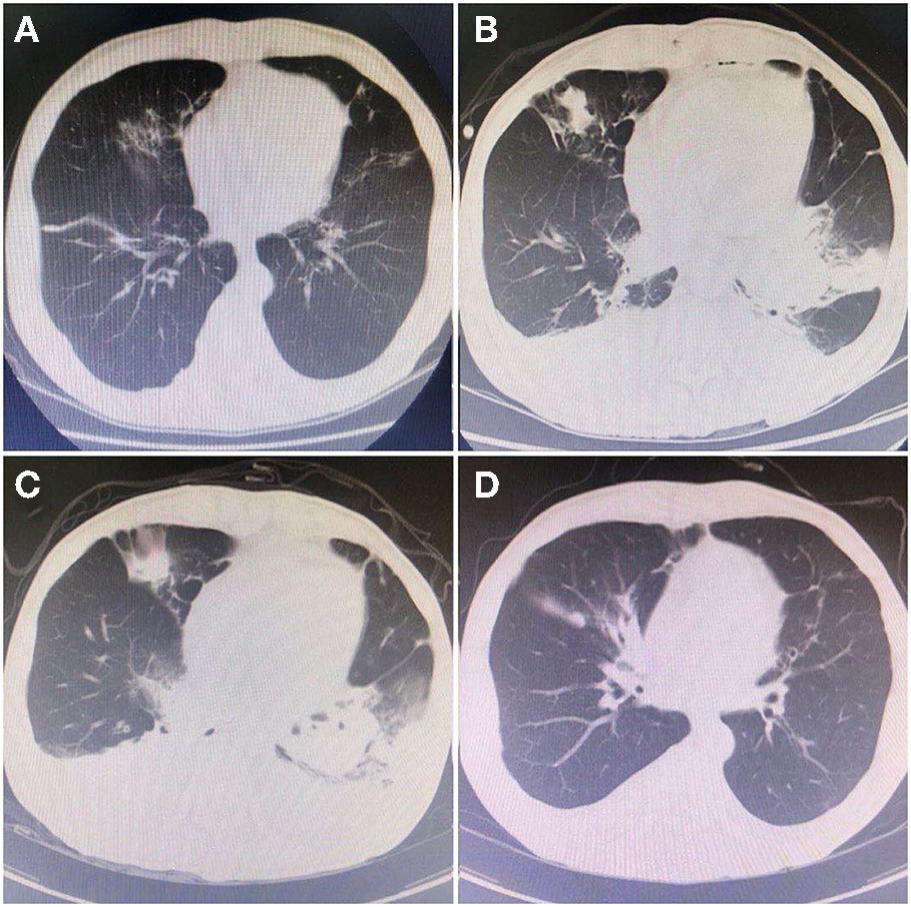
Chest CT indicated dynamic changes during the infections. **(A)** On May 13, 2020, preoperative examination of pulmonary: multiple chronic or old lung lesions. **(B)** On May 31, 2020, the first CT during infection: showed patchy high-density shadows in the basal segment of the lower lobes of both lungs. **(C)** On June 2, 2020, the second CT: range of high-density shadows was more obvious than the previous. **(D)** On June 10, 2020, the last CT: high-density shadows gradually disappeared, which were greatly improving compared with the previous.

A 64-year-old male patient was admitted to the hospital, because of relapsed liver cancer after interventional therapy. The patient met the indications for liver transplantation and was reviewed and approved by the hospital ethics committee to undergo the allogeneic modified piggyback liver transplantation. The operation was successful. The postoperative immunosuppressant regimen was tacrolimus. Liver function gradually recovered, and the patient was making slow progress in the early-stage post-surgery. However, on postoperative day 13, the patient began to experience fluctuating high fevers up to 39°C, especially in the afternoon and morning. Obtaining a sputum sample is by coughing deeply and then spitting the phlegm that comes up into a sample cup during fever for sputum bacterial culture. Preoperative imaging (Figure 1A) showed no significant abnormalities in the lungs. Laboratory examination showed white blood cell (WBC): 14.19^*^10^9^/L, neutrophil% (NE%): 94.2%, procalcitonin (PCT): 1.73 ng/ml, 1–3-β-D Glucan test (G-test): <10 pg/ml (<10 pg/ml), and galactomannan test (GM-test): 0.25 (<0.5), Epstein-Barr virus (EBV) and cytomegalovirus (CMV): negative. Chest Computed Tomography (CT) was obtained showing (Figure 1B): the base of the lower lobe, the right middle lobe, and the left lingual lobe of both lungs were streaked and densely patchy. There was no improvement in fever despite 3 days of empirical cefoperazone/sulbactam treatment. WBC remained elevated, and G-test and GM-test, EBV and CMV test were normal. Chest CT examination showed multiple patchy abnormal shadows in both lungs, which was significantly worse compared with previous chest CT (Figure 1C). After consultation with the Department of Medical Microbiology, it was decided to switch the antibiotic regimen to meropenem and fluconazole. After 2 days of treatment, no improvements were seen. On day 5 of treatment, sputum bacterial culture was reported positive for Nocardia farcinica. According to the symptoms of the patient, repeat chest CT findings, sputum bacterial culture, combining with suggestions from the Department of Medical Microbiology and literatures, the antibiotic regimen was changed to sulfamethoxazole, minocycline, and amikacin. After an additional 5 days, the patient improved, and the fever subsided. Leucocytosis improved and a repeat sputum culture was negative. Chest CT showed (Figure 1D) that multiple abnormal shadows in each lobe disappeared. The patient was discharged from the hospital on the 36th day after the operation. After discharge, the patient was asked to continue oral sulfanilamide and minocycline for 5 months and repeat chest CT imaging regularly.

## Discussion

Liver transplantation is a well-established treatment for end-stage liver disease (1). After a liver transplant, the patient will take immunosuppressants for the life-long, which might result in a hypo-immune state, and increase the risk of infections (2). The genus Nocardia is an aerobic actinomycete, catalase-positive, gram-positive bacillus, with a branching filamentous form first described in 1888 by Edmond Nocard (3). Nocardia sp. is found worldwide in a myriad of environments. Typically, Nocardia is a long-neglected opportunistic pathogen that primarily affects immunosuppressed patients. The disease caused by Nocardia infection can cause a variety of symptoms depending on the site of infection. The main sites of infection include the pulmonary, cutaneous, central nervous system, as well as systemic nocardiosis. Nocardia usually enters the body through the respiratory tract, digestive tract, or broken skin, and can cause a local infection. It can also spread to multiple organs throughout the body through blood circulation, but typically the lungs (5). Cough, sputum, fever, and fatigue are common clinical symptoms. On most occasions, Nocardia infection is very rare but should be considered if tests are negative for other common causes of these symptoms (6). The culture of pathogenic micro-organisms at the site of infection is the gold standard for the diagnosis of Nocardiosis (7). Imaging might help support the history and physical examination which may be related to Nocardia. The common CT findings of Nocardia pneumonia were pulmonary nodules, diffuse or localized lung infiltration, lung abscess, and pleural effusion (8). In many cases, patients with Nocardia are misdiagnosed, leading to high hospital mortality rates. So, if Nocardia infection is suspected, the laboratory must be informed of the suspicion so that the laboratory can take steps to determine the species and antibiotic sensitivity pattern (9). TMP–SMX is the treatment of choice for Nocardial infections. Imipenem, amikacin, and third-generation cephalosporins are also used, and combination therapy can yield better results (10). The treatment duration depends on the infection's location and the patient's immune status. Once diagnosed, treatment of nocardiosis is usually prolonged because of the risk of relapse. For patients with pulmonary involvement, a treatment regimen for 6–12 months is recommended (9). Based on literature review from 2000, 7 cases of Nocardia infection after liver transplant were reported (Table 1) (11–16). We could find that the pulmonary is the most common site of infection, TMP-SMZ is the main first-line option, and patients might be treated for longer periods of up to 6 months.

**Table 1:** Review of 7 cases of Nocardia infection after liver transplantation in PubMed from 2000 to 2020.

**References**	**Publication year**	**Cases**	**Organ(s) involved**	**Treatment**	**Prognosis**
Parra et al. (11)	2008	1	Subcutaneous tissue nodule	TMP-SMZ	Cured
Wiesmayr et al. (12)	2005	1	Pulmonary infiltration	TMP-SMZ	Cured
Marchan et al. (13)	2013	1	Lung, brain,	TMP-SMZ	Cured after
			subcutaneous tissue		6 months
Peleg et al. (14)	2007	2	Pulmonary/bacteremia	TMP-SMZ	Cured
Shin et al. (15)	2007	1	Subcutaneous tissue	TMP-SMZ	Cured after 2 months
Reechaipichitkul et al. (16)	2015	1	Lung, thyroid	TMP-SMZ	Cured after 6 months

## Conclusions

Early diagnosis and targeted antibiotic treatment are critical for Nocardia infections treatment and prognosis. Nocardia infection is very rare but should be considered if the patient has an unexplained fever after liver transplantation.

## Data availability statement

The original contributions presented in the study are included in the article/supplementary material, further inquiries can be directed to the corresponding author/s.

## Ethics statement

The studies involving human participants were reviewed and approved by Ethics Committee of Beijing Chaoyang Hospital. The patients/participants provided their written informed consent to participate in this study. Written informed consent was obtained from the individual(s) for the publication of any potentially identifiable images or data included in this article.

## Author contributions

QH proposed the study. BP and F-FW performed the research and wrote the first draft. BP collected and analyzed the data. All authors contributed to the design, interpretation of the study, to further drafts, and agree to be accountable for the content of the work.

## Conflict of interest

The authors declare that the research was conducted in the absence of any commercial or financial relationships that could be construed as a potential conflict of interest.

## Publisher's note

All claims expressed in this article are solely those of the authors and do not necessarily represent those of their affiliated organizations, or those of the publisher, the editors and the reviewers. Any product that may be evaluated in this article, or claim that may be made by its manufacturer, is not guaranteed or endorsed by the publisher.
